# Chitin Glucan Shifts Luminal and Mucosal Microbial Communities, Improve Epithelial Barrier and Modulates Cytokine Production In Vitro

**DOI:** 10.3390/nu13093249

**Published:** 2021-09-18

**Authors:** Marta Calatayud, Lynn Verstrepen, Jonas Ghyselinck, Pieter Van den Abbeele, Massimo Marzorati, Salvatore Modica, Thibaut Ranjanoro, Véronique Maquet

**Affiliations:** 1 ProDigest BV, Technologiepark 82, 9052 Ghent, Belgium; Lynn.Verstrepen@prodigest.eu (L.V.); Jonas.Ghyselinck@prodigest.eu (J.G.); pieter.vandenabbeele@telenet.be (P.V.d.A.); 2 Center for Microbial Ecology and Technology (CMET), Ghent University, Coupure Links 653, 9052 Ghent, Belgium; 3 KitoZyme SA., Parc Industriel des Hauts Sarts, 4040 Herstal, Belgium; s.modica@kitozyme.com (S.M.); t.ranjanoro@kitozyme.com (T.R.); v.maquet@kitozyme.com (V.M.)

**Keywords:** chitin–glucan, prebiotic, gut microbiome, intestinal barrier, immunomodulation

## Abstract

The human gut microbiota has been linked to the health status of the host. Modulation of human gut microbiota through pro- and prebiotic interventions has yielded promising results; however, the effect of novel prebiotics, such as chitin–glucan, on gut microbiota–host interplay is still not fully characterized. We assessed the effect of chitin–glucan (CG) and chitin–glucan plus *Bifidobacterium breve* (CGB) on human gut microbiota from the luminal and mucosal environments in vitro. Further, we tested the effect of filter-sterilized fecal supernatants from CG and CGB fermentation for protective effects on inflammation-induced barrier disruption and cytokine production using a co-culture of enterocytes and macrophage-like cells. Overall, CG and CGB promote health-beneficial short-chain fatty acid production and shift human gut microbiota composition, with a consistent effect increasing *Roseburia* spp. and butyrate producing-bacteria. In two of three donors, CG and CGB also stimulated *Faecalibacterium prausniitzi*. Specific colonization of *B. breve* was observed in the lumen and mucosal compartment; however, no synergy was detected for different endpoints when comparing CGB and CG. Both treatments included a significant improvement of inflammation-disrupted epithelial barrier and shifts on cytokine production, especially by consistent increase in the immunomodulatory cytokines IL10 and IL6.

## 1. Introduction

During the past few years, published reports have suggested that gut microbiota of the host has been linked to the health status of an individual. The largest pool of microbes occurs in the distal gastrointestinal tract, both in the lumen, where metabolic potency of the microbial ecosystem allows for the fermentation of non-digestible carbohydrates, xenobiotic metabolism, and vitamin and neuropeptide production, as well as in the mucosal niche, where specific microbial communities have a close interaction with host cells [1]. One of the main factors shaping gut microbial communities is the diet. Specifically, dietary fibers are actively fermented by gut microbes leading to the production of short-chain fatty acids [2]. Dietary fiber is composed of carbohydrate polymers undigested and unabsorbed in the human small intestine [3,4,5], including resistant oligosaccharides, non-starch polysaccharides, resistant starches, and lignin [6].

Prebiotics are defined as substrates selectively utilized by host microorganisms, conferring a health benefit [7]. Symbiotics are classified as complementary symbiotics, which must be composed of a probiotic plus a prebiotic, and synergistic symbiotics, for which the substrate is designed to be selectively utilized by the co-administered microorganisms [8]. The beneficial effects of dietary fiber intake on human health have been described, such as a reduced risk for heart disease, stroke, hypertension, specific gastrointestinal disorders, obesity, type 2 diabetes, and some cancers [9,10]. These effects are potentially related to the modulation of the gut microbiota and host metabolism [11]. Although the adequate intake of fiber is associated with many health benefits (25 g/d for healthy adults, European Food Safety Agency—EFSA) [12], the European and USA population is still below recommended levels [6,9].

In this context, dietary supplementation with prebiotic products can be an approach to cover the gap in fiber intake and promote eubiotic gut microbial communities. At the same time, a symbiotic intervention would also benefit the consumer from the complementary and synergistic effect of both pre- and probiotic elements.

The most studied prebiotics are a subset of carbohydrate groups, mainly oligosaccharides [13]. Incorporating novel fibers into the diet can diversify the source of prebiotics for human consumption, also adding value to food industry. In that sense, chitin–glucan is an insoluble fiber composed of chitin (β-1,4-poly-N-acetyl-D-glucosamine) and β-1,3-D-glucan, obtained from *Aspergillus niger* mycelial cell walls, and it is considered a safe food ingredient by the EFSA, with a recommended dose of up to 5 g/day [14].

Human intervention studies have shown the potential health benefits of chitin–glucan intake. Daily consumption of 4.5 g of CG during 6 weeks was associated with reduced oxidized LDL blood levels in a randomized, double-blind, placebo-controlled study (n = 130) [15]. Recently, changes in gut microbiota and derived metabolites have been described in 15 healthy human volunteers consuming 4.5 g/day of CG (3 weeks) [16]. Authors described a specific increase in *Roseburia* and *Eubacterium* genera associated with CG consumption, supporting the results obtained in in vitro and pre-clinical animal models [17,18].

However, the effect of chitin–glucan on the gut mucosal environment and on the host–microbiota interplay, and the potential synergistic effect of chitin–glucan with a probiotic strain has not yet been evaluated.

In this research, we assessed the effect of chitin–glucan (CG) and chitin–glucan plus *Bifidobacterium breve* (CGB) on gut microbiota structure and function, using a colonic fermentation in vitro model simulating the colonic luminal and mucosal compartments of three healthy donors. Supernatants obtained from CG and CGB treatments were subsequently applied to a gut inflammation model of Caco-2 and THP-1 cells to assess the host–microbiota interplay.

## 2. Materials and Methods

### 2.1. Short-Term Colonic Incubations

The objective of these experiments was to determine the effect of chitin–glucan and chitin–glucan and *B. breve* supplementation in vitro on the gut microbial community structure and function from three healthy individuals. The combination of chitin–glucan with *B. breve* was included to compare the effect of chitin–glucan alone (prebiotic) and the potential synergism of a prebiotic and a probiotic strain (symbiotic). Previous literature have shown that *B. breve* can improve the endoscopic scores of inflammatory bowel patients, suggesting a role in immunoregulation [19]. In addition, *B. breve* has potential to reduce body fat in healthy pre-obese individuals [20].

Short-term colonic incubations were performed as previously described in Van den Abbeele et al., [21] with some modifications. Briefly, fresh fecal material (29–35 y, 1 male, 2 females) was collected and independently homogenized in anaerobic phosphate buffer (K_2_HPO_4_ 8.8 g/L; KH_2_PO_4_ 6.8 g/L; sodium thioglycolate 0.1 g/L; sodium dithionite 0.015 g/L) (Chem-lab NV, Zedelgem, Belgium) (1:5 *w*/*v*) using a stomacher bag mixer 10 min (BagMixer 400, Interscience, Louvain-LaNeuve, Belgium). Big particles were removed by centrifugation (2 min, 500 g), and the fecal slurries (10% *v*/*v*) were inoculated in colonic reactors containing CG and CGB and anaerobic sugar-depleted nutritional medium (3.5 g/L K_2_HPO_4_, 10.9 g/L KH_2_PO_4_, 2 g/L NaHCO_3_ (Chem-lab NV, Zedelgem, Belgium), 2 g/L Yeast Extract, 2 g/L peptone (Oxoid, Aalst, Belgium), 0.5 g/L L-cysteine and 2 mL/L Tween80 (Sigma-Aldrich, Bornem, Belgium)) at pH 6.5. In addition, colonic bioreactors included five mucin-coated carriers, prepared according to Van den Abbeele et al., 2013 [22]. Briefly, K1-carriers (AnoxKaldnes AB, Lund, Sweden) were submerged in a mucin dissolution composed by 0.5 g/L gastric porcine mucin type II (Sigma-Aldrich, Bornem, Belgium) and 0.1 g/L bacteriological agar (Oxoid, Aalst, Belgium), combined in a polyethylene netting (Zakkencentrale, Rotterdam, The Netherlands) and submerged in the colonic simulated media.

For each donor, a control condition, a single dose of 5 g/L chitin–glucan (CG/prebiotic), or a single dose of 5 g/L chitin–glucan + *B. breve* (1.4 × 10^7^ colony forming units) (CGB/symbiotic) were tested. CG and CGB were obtained from Kitozyme, Herstal, Belgium. *B. breve* included in CGB was obtained from THT (Gembloux, Belgium). The dose applied in this study was based on the intended intake of CG described as safe by the EFSA [14], and considering the volume of the colon (1 L) of healthy individuals [23].

Incubations were performed in anaerobic conditions for 48 h at 37 °C, 90 rpm and samples were collected at different time points to analyze markers of microbial activity (0, 6, 24, and 48 h) and composition (qPCR of bifidobacteria and *Faecalibacterium prausnitzii* and 16S-targeted Illumina sequencing; 0 and 48 h).

### 2.2. Microbial Metabolic Activity: pH, Gas Production, Short-Chain Fatty Acids (SCFA) and Ammonium

Metabolic activity of the gut microbial communities was assessed by quantifying general markers of fermentation (pH and gas production), lactate and short chain fatty acids.

The pH (Senseline F410; ProSense, Oosterhout, The Netherlands), gas (hand-held pressure indicator CPH6200; Wika, Echt, The Netherlands), lactate (LA), and short-chain fatty acid (SCFA) measurements were performed at 0, 6, 24, and 48 h after starting the colonic incubation. Acetate, propionate, butyrate, and branched SCFAs (isobutyrate, isovalerate, and isocaproate) were measured as described by De Weirdt et al., [24]. According to the manufacturer’s instructions, lactate quantification was performed using a commercial enzymatic assay kit (R-Biopharm, Darmstadt, Germany).

### 2.3. DNA Extraction and 16S RNA Sequencing

DNA was isolated starting from cell pellets from 1 mL sample aliquots (luminal compartment) or 0.25 g sample (mucosal compartment) at 0 and 48 h. Bacterial cells were lysed with 1 mL of lysis buffer [100 mM Tris/HCl pH 8.0, 100 mM EDTA pH 8, 100 mM NaCl, 1% (*m*/*v*) polyvinylpyrrolidone and 2% (*m*/*v*) sodium dodecyl sulphate] and 200 mg of glass beads (0.11 mm, Sartorius, Schaerbeek, Belgium), in a FastPrep^®^- 96 instrument (MP Biomedicals, Santa Ana, CA, USA), two times for 40 s at 1600 rpm. After removing glass beads (5 min, 13,000× *g*), DNA was extracted following a phenol–chloroform extraction. DNA was precipitated with 1 volume ice-cold isopropyl alcohol and 0.1 volume 3 M sodium acetate (1 h, −20 °C). DNA pellet was dried and resuspended in 100 μL 1 × TE (10 mM Tris, 1 mM EDTA) buffer. Samples were diluted in DNAse/RNAse free water to obtain final concentrations of 50 ng/µL and stored at −20 °C. Additionally,16S rRNA gene amplicon sequencing of the V3–V4 region was analyzed at LGC Genomics (Teddington, Middlesex, UK). The 341F (5′-CCTACGGGNGGCWGCAG-3′) and 785R (5′-GACTACHVGGGTATCTAAKCC-3′) primers were used according to [25]. Quality control PCR was conducted using Taq DNA Polymerase with the Fermentas PCR Kit according to the manufacturers’ instructions (Thermo Fisher Scientific, Waltham, MA, USA). The DNA quality was verified by electrophoresis on a 2% (*w*/*v*) agarose gel for 30 min at 100 V. 2.8 and selective quantification of double strand DNA BY Qubit dsDNA High Sensitivity Assay Kit, following manufacturer instructions.

### 2.4. Bioinformatics Analysis of Amplicon Data

The mothur software package (v. 1.39.5) and guidelines were used to process the amplicon data generated by LGC Genomics as previously described in De Paepe et al., 2017 [25]. An operational taxonomic unit (OTU) was defined as a collection of sequences with a length between 402 and 427 nucleotides that are found to be more than 97% similar to one another in the V3–V4 region of their 16S rRNA gene after applying Opticlust clustering [26,27,28,29]. Taxonomy was assigned using the RDP version 16 and silva.nr_v123 database [30,31,32]. The shared file, containing the number of reads observed for each OTU in each sample, was loaded into Microsoft^®^ Excel^®^ 2016 MSO (16.0.11901.20070, Redmond, WA, USA). Reads occurring only 5 times in all samples were removed, as they were supposedly artifacts or bacteria that were not having any biological impact. For the most abundant OTUs, the sequences retrieved from a 3% dissimilarity level fasta file obtained in mothur were classified through the RDP web interface using the RDP SeqMatch tool. The database search was restricted to type strains with only near-full-length and good quality sequences. The sequences were blasted in NCBI against the 16S rRNA gene sequences, selecting only type material, with optimization of the BLAST algorithm for highly similar sequences (accession date: December 2018) [30,32]. Although identification to the species level based on short 300 bp reads may involve some ambiguity, the most likely species classification of a few interesting OTUs is reported in the results sections. In the event of inconsistencies in the results of the RDP SeqMatch tool and NCBI BLAST, no species-level classification is provided.

### 2.5. Microbial Community Analysis by qPCR

qPCR was used to verify microbial changes detected by 16S rRNA sequencing on specific taxa.

Samples collected after 0 and 48 h of incubation were evaluated for the total amount of bifidobacteria and *F. prausnitzii* by qPCR. The DNA was extracted as described in Section 2.3. qPCR assays were performed using a StepOnePlus Real-Time PCR system (Applied Biosystems, Foster City, CA, USA), using the primers and conditions described in Supplementary Tables S1 and S2, adapted from Rinttilä et al., 2004 and Lopez-Siles et al., 2014 [33,34]. Bioline qPCR master mix was obtained from GC Biotech B.V. (Belgium). Each sample was analyzed in technical triplicates and outliers (more than 1 CT difference) were omitted. The samples were checked for correct melt curve peaks. The standard curves for all of the different runs had efficiencies between 90 and 105%. Results are reported as logs (16S rRNA gene copies/mL).

### 2.6. Flow Cytometry

For flow cytometry analysis, 10-fold serial dilutions of luminal and mucosal samples were prepared in anaerobic Dulbecco’s Phosphate-buffered Saline (DPBS) (Sigma-Aldrich, Bornem, Belgium) and stained with 0.01 mM SYTO24 (Life Technologies Europe, Merelbeke, Belgium) for 15′ at 37 °C in the dark. Samples were analyzed on a BD Facsverse (BDBiosciences, Erembodegem, Belgium) using the high-flow-rate setting. Bacteria were separated from medium debris and signal noise by applying a threshold level of 200 on the SYTO channel. Flow cytometry data were analyzed using FlowJo, version 10.5.2.

### 2.7. Cell Culture

Caco-2 cells (HTB-37; American Type Culture Collection) were maintained in Dulbecco’s modified eagle medium (DMEM) containing glucose and glutamine and supplemented with HEPES and 20% (*v*/*v*) heat-inactivated (HI) fetal bovine serum (FBS). THP1-Blue™ NF-κB reporter cells were obtained from InvivoGen (Toulouse, France) and maintained in Roswell Park Memorial Institute (RPMI)1640 medium containing glucose (2 g/L) and glutamine (0.3 g/L), supplemented with HEPES (0.1 mM), sodium pyruvate (1 mM) and 10% (*v*/*v*) HI-FBS. Cells were incubated at 37 °C in a humidified atmosphere of air/CO_2_ (95:5, *v*/*v*).

The Caco-2/THP-1 co-culture was performed as previously described [35]. Briefly, Caco-2 monolayers were cultured for 14 days on 24-well semi-permeable supports, until a functional cell monolayer was obtained. Then, 48 h before the start of the co-culture, THP1-Blue™ cells were seeded in 24-well plates and stimulated for 48 h with phorbol 12-myristate 13-acetate (PMA; 100 nM). After PMA removal, Caco-2 were placed on top of PMA-differentiated THP1-Blue™ cells, followed by apical treatment with complete medium or filter-sterilized (0.22 µm) colonic suspensions collected after 24 h and 48 h of incubation. All treatments were done in biological triplicate. Transepithelial electrical resistance (TEER) was assessed at 0 h and after 24 h of co-culturing, as estimation of the integrity of the epithelial barrier. Further, after 24 h, basolateral medium was removed and THP1-Blue™ were stimulated with ultrapure lipopolysaccharide (LPS) from *Escherichia coli* K12 (InvivoGen, Toulouse, France; 10 ng/mL) or left untreated. After 6 h, basolateral medium was collected for cytokine (Luminex technology, ’s-Hertogenbosch, The Netherlands ) and NF-κB measurements (QUANTI-Blue reagent, InvivoGen, Toulouse, France).

### 2.8. Data Processing and Statistics

All statistical analyses were performed in GraphPad Prism version 8.2.0 for Windows (GraphPad Software, San Diego, CA, USA). All formal hypothesis tests were conducted on the 5% significance level (α = 0.05). Metabolic data, including pH, gas, SCFAs, BCFAs, and lactate were reported as the change between 6 and 0 h (Δ6 h), 24 and 6 h (Δ24 h), and 48 and 24 h (Δ48 h). Descriptive statistics (arithmetic mean and standard error of the mean (SEM)) for all treatment groups were used to summarize the outcomes. Non-paired *t*-tests were used for comparisons of treatments, with Holm-Šídák test for post hoc pairwise comparisons of treatments and control condition.

Then, 16S RNA data (i.e., proportional abundances (%) were multiplied by the absolute cell numbers (cells/mL) obtained via flowcytometry to obtain quantitative data at phylum, family and OTU level. To establish a LOQ for quantitative 16S-targeted Illumina sequencing data, one read was divided by the total amount of reads in each sample, followed by multiplication with the bacterial cell count detected by flow cytometry. This allowed to obtain a LOQ for each sample individually. Quantitative data was analyzed using Calypso software version 8.84, and specific tests were described in the corresponding section.

For cell culture assays, treatment samples were compared to the control samples using two-way ANOVA with Sidak’s multiple comparisons test. Significant differences are represented by (*). (*), (**), (***), and (****) represent *p* < 0.05, *p* < 0.01, *p* < 0.001, and *p* < 0.0001, respectively.

## 3. Results

### 3.1. Chitin–Glucan and Chitin–Glucan + B. breve Affect Fermentation Markers and Induce SCFA Production

**Figure 1:**
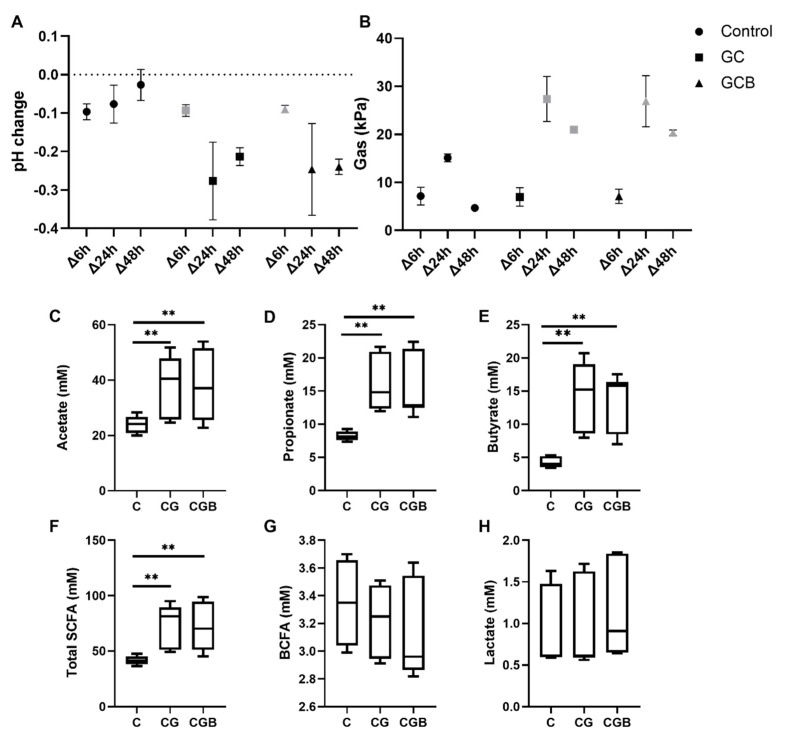
Effect of chitin–glucan and chitin–glucan + *B. breve* on markers of gut microbial metabolism. (**A**) Gas and (**B**) pH change at different time intervals; (**C**–**E**) represent box plots of acetate, propionate, butyrate, total SCFA, BCFA (Δ48 h), and lactate (Δ6 h) change for different treatments. (**A**,**B**) graphs represent mean ± SEM, n = three donors. Box plots represent data from n = three donors with the line at the mean. Statistically significant differences (*p* < 0.05) between treatments and controls are marked with grey symbols in (**A**,**B**) and with asterisks (** *p* < 0.01) in (**C**–**H**).

General markers of microbial fermentation (pH decrease and gas production) were increased in CG- and CGB-treated reactors compared to the control condition (Figure 1A,B). In general, CG- and CGB-treated reactors showed an increase in SCFAs at 48 h (Δ48 h; *p* < 0.05), with fold changes to control of approximately 1.6, 2, 3 and 1.7 for acetate, propionate, butyrate, and total SCFA, respectively (Figure 1C–F). Overall, propionate and butyrate did not show significant differences between CG and CGB treatments (Supplementary Figure S1).

When analyzing each donor independently, donors 1 and 3 had the highest response to CG and CGB in total SCFA production (two-fold change to control), with the highest butyrate production observed in donor 1 (CG = 4.8 ± 0.4 and CGB = 4.1 ± 0.3-fold change to control) and the highest propionate increase shown in donor 3 (CG = 2.7 ± 0.1 and CGB = 2.8 ± 0.1-fold change to control). Overall, donor 2 was the least responsive to CG and CGB.

BCFA and lactate showed high interindividual variability, and when considering the three donors together, no significant differences were observed compared to control (Figure 1G,H). When looking at each donor, lactate increased compared to the control in the CGB-exposed group (Δ6 h, *p* < 0.05) (Supplementary Table S3), with donor 1 showing the highest level. BCFAs were reduced in donor 2 and 3 CG-treated reactors and CGB-treated reactors (Supplementary Table S4).

### 3.2. CG and CGB Modify Microbiota Structure at the Luminal and Mucosal Level

The impact of CG and CGB supplementation on gut microbial structure was analyzed by comparing treated reactors to control reactors containing the same background nutritional media without any treatment.

In the luminal compartment, CG and CGB increased Chao1 index (*p* = 0.044) (Supplementary Figure S2), whereas in the mucosal compartment, no significant effects were observed for alpha diversity indices, richness or evenness (Supplementary Figure S3).

**Figure 2:**
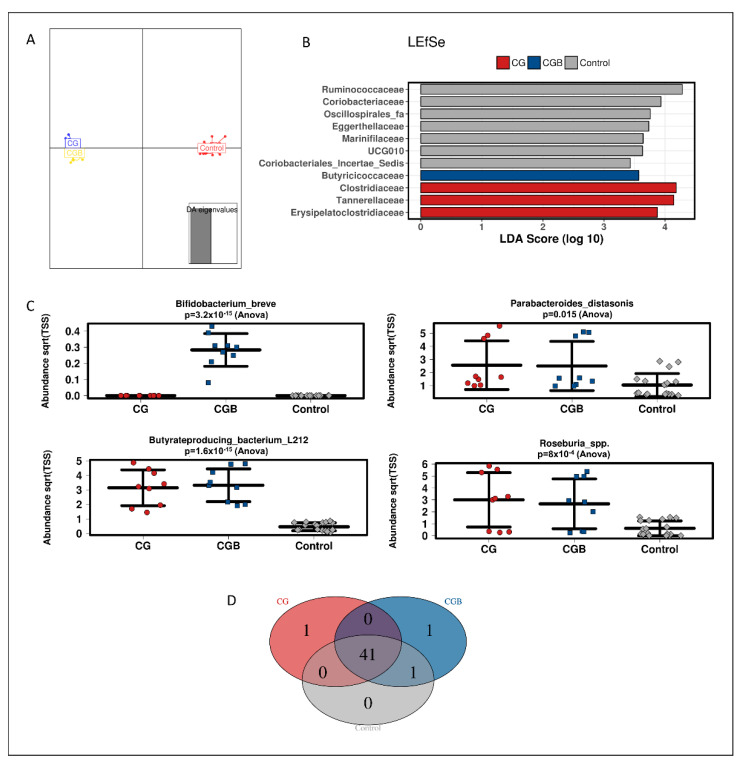
Effect of GC and GCB treatments on luminal microbial composition. (**A**) Discriminant analysis of principal components (DAPC) plot of the luminal compartment at family level; (**B**) linear discriminant analysis effect size (LEfSe) at family level; (**C**) strip charts of selected features at OTU level; (**D**) core microbiota at OTU level. Graphs represent 16S rRNA sequencing data from three healthy individuals (n = 3) in technical triplicates (n = 3).

At family level, a discriminant analysis of principal components (DAPC) showed a different clustering of CG and CGB to control (*p* = 0.008, Adonis test based on Bray–Curtis distance) (Figure 2A). To determine which features could explain the differences between clusters, we used linear discriminant analysis of effect size (LEfSe). LEfSe analysis showed an enrichment of *Butyriciococcaceae* in CGB-treated reactors and *Clostridiaceae*, *Tannerellaceae* and *Erysipelatoclostridiaceae* in CG-treated reactors (LDA score > 3, Figure 2B).

At OTU level, *Butyrate-producing bacterium-L212* (OTU7), *Parabacteroides diastonis* (OTU12/45) and *Roseburia* spp. (*R. hominis* and *R. inulinivorans*; OTU1/OTU2) were enriched by CG and CGB (Figure 2C). *B. breve* (related to OTU211) was increased by CGB treatment. *B. breve* was a unique feature for the CGB treatment (Figure 2D), indicating the absence of this specific strain in the microbial background community of the three different donors. *Clostridium symbiosum* (OTU56), was a unique feature of CGB treatment (Figure 2D).

When analyzing each individual independently, CG increased *Rikenellaceae* families 1 and 3, whereas in donor 3, *Muribaculaceae, Prevotellaceae*, and unclassified members of the class Bacteroidia were also enriched (Supplementary Table S5). Co-supplementation of the probiotic with chitin–glucan did not further stimulate Bacteroidetes phylum members in any of the donors.

**Figure 3:**
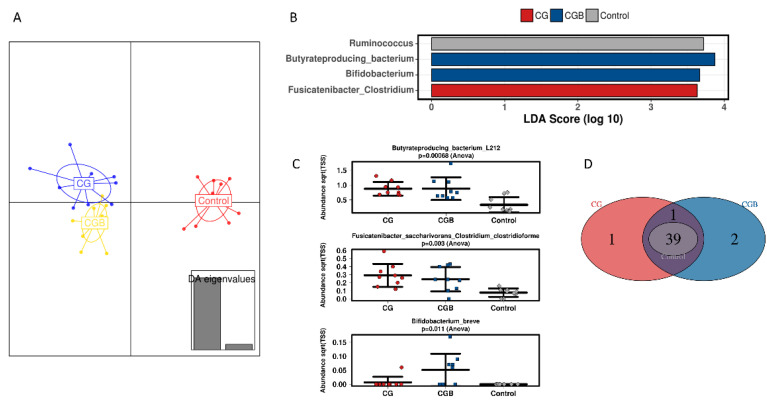
Effect of GC and GCB treatments on mucosal microbial composition. (**A**) Discriminant analysis of principal components (DAPC) plot of the mucosal compartment at family level; (**B**) core microbiota at OTU level; (**C**) linear discriminant analysis effect size (LEfSe) at family level; (**D**) selected significant different features at OTU level between control and treatments. Graphs represent 16S rRNA sequencing data from three healthy individuals (n = 3) in technical triplicates (n = 3).

At the mucosal level, different treatments had less effect than in the luminal compartment. At family level, no significant effect was observed (Adonis test based on Bray–Curtis distance, *p* = 0.847), although the DAPC plot shows a different clustering between treatments and control (Figure 3A). At OTU level, *B. breve* (OTU7), *Butyrate-producing bacterium*-L212 (OTU12) and *Fusicatenibacter saccharivorans/Clostridium clostridioforme* (OTU18) were enriched in CGB-treated reactors, (Figure 3B,C). Two unique features were found in the mucosal environment of the CGB treatment, corresponding to *B. breve* (OTU7) and *B. bifidum* (OTU21). *Unclassified_Firmicutes* (OTU91) was unique for the CG treatment, while *Coprococcus catus* (OTU58) was only detected in CG and CGB, but not in the control condition (Figure 3D).

Interindividual differences were also observed in the mucosal compartment with a mild stimulatory effect of CG on *Bifidobacterium adolescentis/B. faecale* (OTU34) in donor 3. In donors 1 and 2, CG stimulated *Bacteroidaceae* family, corresponding to Bacteroides *uniformis* (OTU8 and OTU11) (Supplementary Tables S6 and S7). OTU9, associated with a member of the *Lachnospiraceae* NK4A136 group, was enriched in donors 1 and 3, while *Eubacteriaceae* and *Eryspelatoclostridiaceae* members were stimulated in donors 2 and 1, respectively (Supplementary Tables S6 and S7).

### 3.3. Specific Modulation of Bifidobacteria and F. prausnitzii by CG and CGB Based on qPCR Quantification

**Figure 4:**
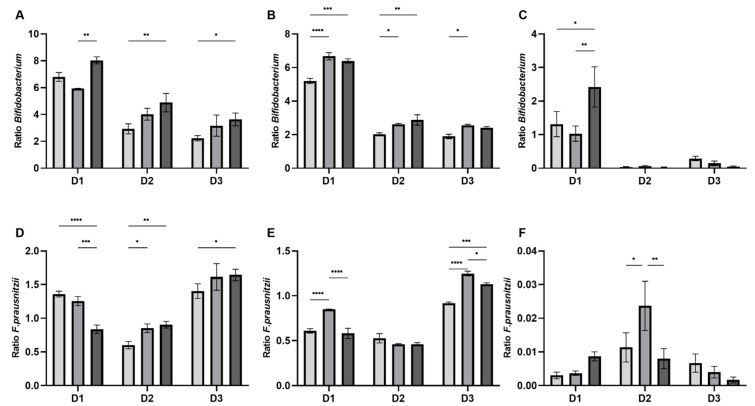
Effect of CG and CGB on specific members of the microbial community. Bars represent the ratio between bifidobacteria (**A**–**C**) or *F. prausnitzii* (**D**–**F**) qPCR counts (copies/mL) in control, CG and CGB conditions respect to time 0 levels. Each donor is represented in the X axis (D1, D2, D3; mean ± SEM; n = 3). Graphs (**A**,**D**) and graphs (**B**,**E**) represent the luminal compartment at 24 h and 48 h, respectively. Graphs C and F represent the mucosal compartment at 48 h. Statistically significant differences were calculated using of bifidobacterial or *F. prausnitzii* between control and treatments (*p* < 0.05, two-way ANOVA) and are marked with asterisks (* *p* < 0.05; ** *p* < 0.01; *** *p*< 0.001; **** *p* < 0.0001).

The effect of different treatments on bifidobacteria and *F. prausnitzii* was donor-dependent. In the luminal compartment, CG-treated reactors showed a higher ratio of *Bifidobacterium* at 48 h compared to T0, with consistent results for the three donors (Figure 4A,B). At 24 h, CGB increased the ratio of *Bifidobacterium* in all the donors, and only in donor 1 and donor 2 at 48 h. Mucosal colonization with *Bifidobacterium* was only affected by CGB in donor 1 at 48 h (Figure 4C).

*F. prausnitzii* ratio in the luminal compartment at 24 h was increased in donor 2 by CG and CGB treatments and in donor 3 only by CGB, while in donor 1, the opposite trend was observed for both conditions (Figure 4D). At 48 h, donor 1 showed a different response, with increases in *F. prausnitzii* ratio by CG. Similarly, *F. prausnitzii* ratio was also increased in donor 3 by CG but also by CGB treatment (Figure 4E).

In the mucosal compartment, only donor 2 showed an increased *F. prausnitzii* ratio, despite the low levels (<0.05) (Figure 4F).

### 3.4. Fecal Supernatants from CG and CGB Colonic Fermentations Improved Inflammation-Induced Damage to the Intestinal Epithelium and Modulate Cytokine Production

The effect of filter-sterilized samples from CG and CGB reactors on epithelial barrier function and modulation of cytokine production was tested in a gut inflammation model.

**Figure 5:**
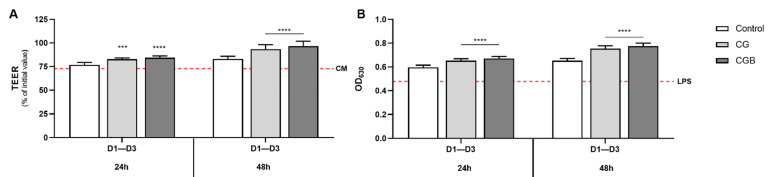
Effect of simulated colonic fluids exposed to CG and CGB during 24 h and 48 h on transepithelial electrical resistance (**A**) and NF-κB activation (**B**) in a simulated colonic model. Bars represent the mean ± SEM (n = 9). Significant differences between control condition and different treatments are represented by (*** *p* < 0.001) and (**** *p* < 0.0001). CM = cell culture media (negative control); LPS = lipopolysaccharide (positive control).

Cells exposed to colonic simulated supernatants supplemented with CG and CGB for 24 and 48 h showed higher TEER values than the control condition (*p* < 0.05). (Figure 5A).

Interindividual differences in TEER response are shown in Supplementary Figure S4. The highest response was observed in donor 3 after exposure to CG (110.6 ± 1.3%) and CGB (113.1 ± 1.9%) 48 h supernatants.

In general, all colonic batch suspensions increased the NF-κB activity above the positive control (LPS). Concretely, cells exposed to 24 h and 48 h CG supernatants showed higher NF-κB activity values compared to the control condition (Figure 5B). GCB supernatants also induced an increase in NF-κB at 24 h and 48 h (Figure 5B). Interindividual differences in NF-κB response to CG and CGB supernatants are presented in Supplementary Figure S5.

**Figure 6:**
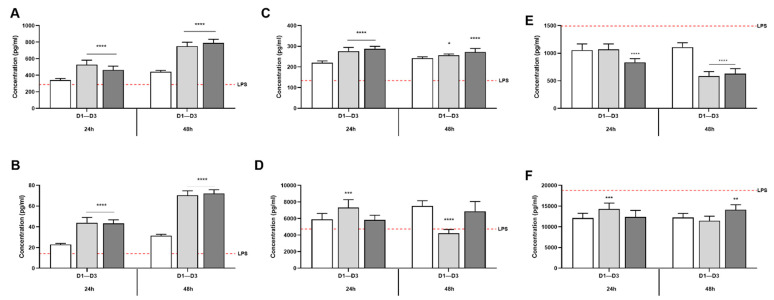
Effect of simulated colonic fluids exposed to CG and CGB during 24 h and 48 h on cytokine production. Bars represent the mean (pg/mL) ±SEM, n = 9 of IL6 (**A**), IL10 (**B**), IL1β (**C**), TNFα (**D**), CXCL10 (**E**), and MCP1 (**F**). Significant differences between control condition and different treatments are represented by (*). (*), (**), (***), and (****) represent *p* < 0.05, *p* < 0.01, *p* < 0.001, and *p* < 0.0001, respectively. LPS = lipopolysaccharide (positive control).

CG and CGB had a significant effect on the production of cytokines in the inflamed simulated epithelium (Figure 6). Compared to the LPS+ control, both CG and CGB 24 h-colonic suspensions increased IL6, IL10 and IL1β levels compared to their controls (*p* < 0.05) (Figure 6A–C). For IL6 and IL10, this effect was most pronounced in cells exposed to 48 h colonic suspension (Figure 6A,B), without significant differences between CG and CGB treatments. For IL1β, donor-specific responses were observed in cells exposed to 48 h colonic incubations (Supplementary Figure S6). TNFα levels were induced by 24 h CG colonic supernatants compared to control (*p* < 0.05), while 48 h CG colonic supernatants reduced TNFα levels (Figure 6D). When considering all donors together, CGB did not show significant differences in TNFα levels; however, specific trends for each donor can be observed in Supplementary Figure S6. Concretely, CG treated samples from donor 3 significantly increased the TNF-α secretion compared to the control. Samples of 48 h of colonic fermentation of both CG and CGB reduced secretion of TNF-α in donors 1 and 3, while in donor 2, CGB significantly increased the TNF-α secretion.

CXCL10 levels were reduced by 24 h CGB supernatants compared to control condition (*p* < 0.05). After 48 h of fermentation, both CG and CGB supernatants reduced CXCL10 levels compared to the control (*p* < 0.05) (Figure 6E). The highest inhibitory effect on CXCL10 production was observed for donors 1 and 3 (Supplementary Figure S6).

Overall production of MCP1 was increased by 24 h CG and 48 h CGB colonic supernatants compared to the control (*p* < 0.05) (Figure 6F). In donors 2 and 3, 24 h CGB treatment significantly decreased the MCP-1 secretion. After 48 h of colonic fermentation, both CG and CGB treatment significantly decreased MCP-1 secretion in donor 3 (Supplementary Figure S6).

## 4. Discussion

This study showed that CG and CGB supplementation modulate microbial activity, mucosal colonization and cytokine production in vitro, with consistent results across donors in improving the intestinal epithelial barrier, inducing anti-inflammatory IL10 production and promoting butyrate-producing bacteria colonization of the mucosal niche.

A single dose (5 g/L) of CG and CGB increased total short-chain fatty acid production, with a consistent effect on the three tested donors. Concretely, health-promoting butyrate and propionate were stimulated by both treatments. These SCFAs are involved in regulating host energy metabolism and preserving mucosal integrity, with both intestinal and extra-intestinal effects [36,37]. Butyrate is a primary energy source for colonocytes and supports intestinal homeostasis through anti-inflammatory activity, while propionate modulates the secretion of GLP-1 and PYY by enteroendocrine cells in the gut [38]. Acetate also has a significant function on intestinal homeostasis, regulating gut pH, acting as an energy substrate for colonocytes, having a significant impact on host metabolism, and playing a role in preventing intestinal infections [39,40]. Remarkably, inflammatory bowel disease (IBD) patients have lower SCFAs levels in feces than healthy controls, together with reduced levels of SCFAs-producing bacteria, including *F. prausnitzii* and *Roseburia intestinalis*, in intestinal mucosa and feces [41]. The effect of CG on increasing SCFAs may be beneficial for improving intestinal health in specific dysbiotic conditions. In that sense, shifts in microbial communities observed in our in vitro study are in line with results from human interventions and in vivo animal studies using CG [16,18]. Rodriguez et al., 2020 showed increases in *Roseburia* spp. after a 3 week intervention in humans with 4.5 g CG/day. In our study, changes in microbiota composition were already observed after 48 h of treatment, indicating a quick modulatory activity of CG towards healthy microbial ecosystems, with specific increase in *Roseburia* spp. related to *R. hominis* and *R. inulinivorans*.

We incorporated the mucosal environment in the in vitro setup to obtain a better understanding of prebiotic mechanisms at the host interface. The close interplay between gut epithelium and mucosal microbial communities has been described as a key factor for immune modulation, intestinal maturation, and competitive exclusion of pathogens [42,43,44]. The mucosal ecosystems have a different structure from the luminal milieu, including specific microorganism assembly [43,44]. We observed the colonization of the mucosal niche by *B.breve* and *B. bifidum* in CGB treated-reactors, the last potentially present as commensal in the fecal background microbiota. Schroeder et al., 2019 suggested that *Bifidobacterium* strains prevent intestinal disease by modulating the function of the mucus layer [45]. Concretely, several human disorders related to autoimmunity and dysregulated pro-inflammatory paths have been linked to a reduction in the abundance of mucosal *F. prausnitzii* and *Coprococcus* spp. [46]. From our results, *Coprococcus* spp. was only present in the mucosal communities of CG and CGB treatments and not in the control condition. Mucins also serve as a growth substrate for butyrate-producing bacteria, possibly via cross-feeding with mucin-degrading microbes such bifidobacteria that deliver partial breakdown products, acetate and/or lactate [47,48]. The main difference induced in the mucosal structure between CG and CGB was related to the presence of *B. breve* as a unique feature in CGB treatments, suggesting a minor impact of the probiotic strain compared to CG on background microbiota. Similarly, the effect of CG on microbial metabolism was not modified with the presence of *B. breve*, suggesting that the prebiotic activity of CG has a major role in modulating the microbial activity, while *B. breve* did not support a synergistic effect.

Based on 16S RNA data, GC and GCB increased a butyrate-producing bacterium-L212 in luminal and mucosal compartments, while *F. prausnitzii* ratio was increased by both treatments, especially in the luminal compartment. *F. prausnitzii* has been previously described as an indicator of intestinal health and a key commensal microorganism with immunomodulatory properties [49,50].

In that sense, we observed that filter-sterilized fecal supernatants from CG and CGB improved epithelial barrier damage in an inflammatory intestinal model, and increased IL10 and IL6 production while reducing proinflammatory TNFα and CXCL10. The impaired epithelial barrier has been associated with inflammatory pathological conditions of the gut and restoration of intestinal function has been proposed as a potential therapeutic target to promote recovery of intestinal homeostasis [51]. Recently, a microbial anti-inflammatory molecule (MAM) produced by *F. prausnitzii* efficiently re-established the intestinal barrier structure via the regulation of the tight junction pathway and ZO-1 expression [52], while previous studies also showed that *F. prausnitzii* is associated with anti-inflammatory effects via IL10 induction in dendritic cells [53]. *Roseburia intestinalis* and bifidobacteria have also been described as effective commensals in promoting intestinal barrier function [54,55,56]. In that sense, MAM has also been described as a down regulator of NFkβ [55], which, in our study, was increased by filter-sterilized supernatants derived from CG and CGB fermentations. Linked to cytokine production, NFkβ signaling is also responsible for coordinating inflammatory and immune responses, also participating in the control of cell proliferation and survival and maintaining epithelial barrier function [57]. In our model, NFkβ activation is enhanced upon butyrate treatment, linked to the expression of the immunomodulatory cytokines IL-6 and IL-10 [21]. CG and CGB similarly increase NFkβ activity, potentially modulating cytokine production. Microbial metabolites, such as butyrate, acetate, or propionate, and unknown molecules may also be involved in the response observed in our model. For example, butyrate has been shown to enhance IL-10 secretion in human monocytes. IL-10 is a central cytokine in maintaining mucosal tolerance, signaling different lymphocyte T subpopulations, and influencing selective colonization of the gut [58], and it has been proposed as a target for alleviating gut inflammatory conditions [59]. Moreover, *Roseburia intestinalis* derived flagellin has been proved as an effective modulator of inflammatory gut responses [60]. In addition, fecal supernatants derived from chitin–glucan fermentation had a stimulatory effect on IL1β production while reducing CXCL10, suggesting that they have a significant modulatory role in cytokine production. Further assessment including more donors and testing specific signaling pathways are needed.

Despite the current limitations of this study, such as the limited number of donors, the microbiota analysis based on the taxonomic profile via 16S rRNA gene sequencing, and lack of characterization of the metabolic activity in the mucosal niche, we proved a mechanistic link between CG and CGB, gut microbiota modulation, and intestinal health. The potential of CG and CGB for balancing the anti- and pro-inflammatory status and improving epithelial barrier deserves further investigation in human populations.

## 5. Conclusions

Using a combination of complex in vitro tools mimicking the gut microbiota from the mucosal and luminal environment and the host intestinal barrier, we demonstrated that CG and CGB supplementation could consistently stimulate *Roseburia* spp. and butyrate-producing bacteria while inducing a significant improvement of the inflammation-disrupted epithelial barrier and modulated cytokine production. *B. breve* colonized both, luminal and mucosal compartment. Incorporating the mucosal niche provides novel information on potential interactions between pre- and pro-biotic combinations and complex microbial communities.

## Data Availability

Data supporting reported results can be obtained by direct request to corresponding author (M.M.)

## Supplementary Materials

The following are available online at https://www.mdpi.com/article/10.3390/nu13093249/s1, Table S1: Primers used to quantify bifidobacteria, and *Faecalibacterium prausnitzii* by qPCR. Table S2: Conditions used to quantify bifidobacterial and *Faecalibacterium prausnitzii* by qPCR. Table S3: Effect of CG and CGB on lactate change (Δ6 h) for three independent donors. Data represent mean ± SEM of n = 3 technical replicas. Statistically significant differences (*p* < 0.05, unpaired *t*-test) between treatments and control are marked in bold. Table S4: Effect of CG and CGB on BCFA change (Δ48 h) for three independent donors. Data represent mean ± SEM of n = 3 technical replicas. Statistically significant differences (*p* < 0.05, unpaired *t*-test) between treatments and control are marked in bold. Table S5: Average abundances (log (cells/mL)) of the bacterial families in the luminal environment in the different test conditions (Ctrl = control, CG = chitin–glucan, CGB = chitin–glucan + *B. breve*) after 48 h. Statistically significant differences are indicated in bold. The intensity of shading is correlated with the abundance of a given family under different conditions per donor. Significant differences reported in the tables (bold values) refer to differences CG versus the control condition and to CG versus CGB. Table S6: Average relative abundances (%) of the bacterial families in the mucosal environment in the different test conditions (Ctrl = control, CG = chitin–glucan, CGB = chitin–glucan + *B. breve*) after 48 h. Statistically significant differences are indicated in bold. The intensity of shading is correlated with the abundance of a given family under different conditions per donor. Significant differences reported in the tables (bold values) refer to differences CG versus the control condition and to CG versus CGB. Table S7: Average relative abundances (%) of the most abundant OTUs in the mucosal environment in the different test conditions (Ctrl = control, CG = chitin–glucan, CGB = chitin–glucan + *B. breve*) after 48 h. Statistically significant differences are indicated in bold. The intensity of shading is correlated with the abundance of a given family under different conditions per donor. Significant differences reported in the tables (bold values) refer to differences CG versus the control condition and to CG versus CGB. Figure S1: Effect of CG and CGB on SCFA and BCFA in different donors. Figure S2: Diversity, richness and evenness in the luminal compartment. Figure S3: Diversity, richness and evenness in the mucosal compartment. Figure S4: Interindividual differences of TEER in response to CG and CGB. Figure S5: Interindividual differences of NFkβ in response to CG and CGB. Figure S6: Interindividual differences of cytokine production in response to CG and CGB.
