# Supplementary material for: Chitin Glucan Shifts Luminal and Mucosal Microbial Communities, Improve Epithelial Barrier and Modulates Cytokine Production In Vitro
Source: Nutrients. 2021 Sep 18;13(9):3249. doi: 10.3390/nu13093249 (PMC8467507; doi:10.3390/nu13093249)
Supplement: Supplementary file 1 [file nutrients-13-03249-s001.zip › nutrients-1355053-supplementary.pdf]

Supplementary materials

Supplementary tables

Supplementary table S1. Primers used to quantify Bifidobacteria, and Faecalibacterium prausnitzii by qPCR.

**Targeted group**	**Primer name**	**Sequence (5' – 3')**	**Reference**
Bifidobacterium spp.	Bif243F	TCGCGTCYGGTGTGAAAG	*(Rinttilä et al., 2004)*
	Bif243R	CCACATCCAGCRTCCAC	
Faecalibacterium prausnitzii	Fpra 428F	TGTAAACTCCTGTTGTTGAGGAAGATAA	*(Lopez-Siles et al., 2014)*
	Fpra 583R	GCGCTCCCTTTACACCCA	

Supplementary table S2. Conditions used to quantify bifidobacterial and Faecalibacterium prausnitzii by qPCR.

Program	Cycle	Temperature (°C)	Heating (hh:mm:ss)	Ramp (°C/s)
Pre-incubation	1	95	00:10:00	1.6
Amplification	40	95	00:00:15	1.6
		60	00:00:30	1.55
		72	00:00:30	1.6
Melting curves	1	95	00:00:15	1.6
		60	00:01:00	1.55
		75	00:00:15	0.075

Supplementary table S3. Effect of CG and CGB on lactate change (Δ6h) for three independent donors. Data represent mean ± SEM of n=3 technical replicas. Statistically significant differences ($p < 0.05$, unpaired t-test) between treatments and control are marked in bold.

	Control			CG			CGB		
				mM Lactate Δ6h					
**D1**	1.53	±	0.12	1.66	±	0.08	**1.84**	±	**0.01**
**D2**	0.59	±	0.01	0.60	±	0.01	**0.65**	±	**0.01**
**D3**	0.62	±	0.03	0.60	±	0.05	**0.90**	±	**0.02**

Supplementary table S4. Effect of CG and CGB on BCFA change (Δ48h) for three independent donors. Data represent mean ± SEM of n=3 technical replicas. Statistically significant differences ($p < 0.05$, unpaired t-test) between treatments and control are marked in bold.

	Control			CG			CGB		
				BCFA mM Δ48h					
**D1**	3.38	±	0.14	3.23	±	0.15	**2.94**	±	**0.15**
**D2**	3.02	±	0.04	**2.93**	±	**0.03**	2.91	±	0.07
**D3**	3.67	±	0.04	**3.49**	±	**0.03**	3.58	±	0.09

Supplementary table S5. Average abundances (log(cells/ml)) of the bacterial families in the luminal environment in the different test conditions (Ctrl= control, CG= chitin-glucan, CGB= chitin-glucan + B. breve) after 48h. Statistically significant differences are indicated in bold. The intensity of shading is correlated with the abundance of a given family under different conditions per donor. Significant differences reported in the tables (bold values) refer to differences CG versus the control condition and to CG versus CGB.

Phylum	Family	Lumen								
		Donor 1			Donor 2			Donor 3		
		Ctrl	CG	CGB	Ctrl	CG	CGB	Ctrl	CG	CGB
Actinobacteria	*Atopobiaceae*	<LOQ	<LOQ	<LOQ	<LOQ	<LOQ	<LOQ	6,36	6,49	6,43
	Bifidobacteriaceae	6,76	6,71	6,89	6,92	**6,64**	6,82	8,13	**8,01**	**8,16**
	Coriobacteriaceae	7,26	7,14	7,12	7,85	7,68	7,62	7,70	7,50	7,47
	Coriobacteriales Incertae Sedis	<LOQ	<LOQ	<LOQ	<LOQ	<LOQ	<LOQ	5,88	**5,55**	<LOQ
	Coriobacteriales unclassified	<LOQ	<LOQ	<LOQ	<LOQ	<LOQ	<LOQ	5,79	5,55	5,94
	Eggerthellaceae	6,92	6,85	6,83	6,92	**6,55**	6,58	6,46	6,59	6,50
Bacteroidetes	*Bacteroidaceae*	8,38	**8,97**	9,00	8,67	**8,96**	8,97	8,74	8,62	8,72
	Bacteroidales unclassified	5,47	5,85	5,79	<LOQ	**5,88**	5,79	6,20	7,08	6,81
	Bacteroidia unclassified	6,37	**6,66**	6,50	<LOQ	<LOQ	<LOQ	5,88	**6,59**	6,33
	Barnesiellaceae	5,77	5,96	6,00	6,20	5,98	5,88	5,52	5,65	<LOQ
	Marinifilaceae	6,40	6,31	**6,04**	6,64	**5,82**	5,72	6,46	6,19	5,86
	Muribaculaceae	<LOQ	<LOQ	<LOQ	<LOQ	<LOQ	<LOQ	6,61	**6,88**	7,03
	Prevotellaceae	<LOQ	<LOQ	<LOQ	<LOQ	<LOQ	<LOQ	5,49	**8,94**	8,94
	Rikenellaceae	6,97	**7,25**	7,06	7,49	7,36	7,21	6,62	**6,99**	7,09
	Tannerellaceae	7,52	**8,19**	**8,12**	7,29	**7,57**	7,49	8,15	**9,02**	9,07
Firmicutes	*Acidaminococcaceae*	7,11	**7,79**	7,69	6,91	**7,37**	7,30	<LOQ	<LOQ	<LOQ
	Anaerofustaceae	<LOQ	<LOQ	<LOQ	4,97	5,18	<LOQ	5,72	**<LOQ**	5,53
	Anaerovoracaceae	6,31	6,18	6,24	5,69	5,76	**5,16**	7,01	7,12	7,08
	Bacillaceae	<LOQ	<LOQ	<LOQ	<LOQ	<LOQ	<LOQ	<LOQ	5,58	<LOQ
	Butyricicoccaceae	5,92	6,20	6,05	6,12	6,04	5,84	6,22	6,01	6,29
	Christensenellaceae	6,08	6,10	6,01	<LOQ	<LOQ	<LOQ	6,95	**7,28**	7,28
	Clostridia UCG-014	5,71	5,48	5,52	<LOQ	<LOQ	<LOQ	7,23	7,18	7,24
	Clostridia vadinBB60 group	<LOQ	<LOQ	<LOQ	<LOQ	<LOQ	<LOQ	5,17	<LOQ	<LOQ
	Clostridiaceae	6,39	7,53	7,48	5,77	**7,53**	7,32	6,47	6,53	6,58
	Defluviitaleaceae	<LOQ	<LOQ	<LOQ	<LOQ	<LOQ	<LOQ	5,52	5,55	<LOQ
	Enterococcaceae	8,61	**8,46**	8,47	6,80	6,69	6,66	<LOQ	<LOQ	<LOQ
	Erysipelatoclostridiaceae	<LOQ	**7,49**	7,42	5,79	**7,78**	7,61	5,45	6,16	6,13
	Erysipelotrichaceae	5,87	6,10	6,13	5,24	<LOQ	<LOQ	6,84	**7,79**	8,10

	Eubacteriaceae	6,87	6,79	7,04	<LOQ	<LOQ	<LOQ	7,60	7,44	7,53
	Firmicutes unclassified	6,14	<LOQ	<LOQ	<LOQ	<LOQ	<LOQ	<LOQ	<LOQ	<LOQ
	Lachnospiraceae	8,39	**9,38**	9,32	8,64	**9,15**	**9,08**	8,31	**8,96**	9,04
	Lactobacillales unclassified	6,13	5,87	6,03	<LOQ	<LOQ	<LOQ	<LOQ	<LOQ	<LOQ
	Monoglobaceae	<LOQ	<LOQ	<LOQ	4,89	<LOQ	<LOQ	5,92	6,18	5,93
	Oscillospiraceae	7,59	7,66	7,53	7,47	7,41	7,37	8,13	8,28	8,31
	Oscillospirales	<LOQ	<LOQ	<LOQ	5,36	5,15	5,33	5,55	<LOQ	<LOQ
	Oscillospirales unclassified	<LOQ	<LOQ	<LOQ	<LOQ	<LOQ	<LOQ	6,10	**<LOQ**	<LOQ
	Peptococcaceae	5,79	5,91	**5,39**	<LOQ	<LOQ	<LOQ	5,98	5,58	5,73
	Peptostreptococcaceae	6,65	**7,05**	7,05	5,77	5,80	5,96	6,23	6,25	6,46
	Peptostreptococcales-Tissierellales unclassified	5,55	5,46	<LOQ	<LOQ	<LOQ	<LOQ	<LOQ	<LOQ	<LOQ
	RF39	<LOQ	<LOQ	<LOQ	<LOQ	<LOQ	<LOQ	5,70	5,73	5,78
	Ruminococcaceae	7,14	7,29	7,33	6,27	6,12	6,00	8,02	7,98	7,98
	Selenomonadaceae	<LOQ	<LOQ	<LOQ	<LOQ	<LOQ	<LOQ	<LOQ	<LOQ	<LOQ
	Staphylococcaceae	<LOQ	<LOQ	<LOQ	<LOQ	<LOQ	<LOQ	<LOQ	5,81	<LOQ
	Streptococcaceae	5,53	6,96	6,78	5,76	5,25	5,53	5,17	<LOQ	<LOQ
	UCG-010	5,27	<LOQ	<LOQ	<LOQ	<LOQ	<LOQ	5,52	5,44	<LOQ
	Veillonellaceae	6,13	6,50	6,40	7,83	**7,38**	7,43	7,26	**8,24**	8,30
Verrucomicrobia	*vadinBE97*	5,24	<LOQ	<LOQ	<LOQ	<LOQ	<LOQ	5,41	5,61	<LOQ
Proteobacteria	*Alphaproteobacteria unclassified*	<LOQ	<LOQ	<LOQ	<LOQ	<LOQ	<LOQ	5,65	5,53	<LOQ
	Desulfovibrionaceae	6,40	**6,91**	6,79	7,15	**6,56**	6,55	6,50	6,41	6,63
	Enterobacterales unclassified	<LOQ	<LOQ	<LOQ	<LOQ	<LOQ	<LOQ	5,13	<LOQ	<LOQ
	Enterobacteriaceae	8,32	8,41	8,34	7,57	7,20	7,16	8,61	8,55	8,64
	Gammaproteobacteria unclassified	5,27	<LOQ	<LOQ	<LOQ	<LOQ	<LOQ	<LOQ	<LOQ	<LOQ
	Sutterellaceae	6,60	**6,87**	**6,77**	7,52	7,39	7,33	7,09	6,97	6,91
Unclassified	*Bacteria unclassified*	<LOQ	<LOQ	5,31	<LOQ	<LOQ	<LOQ	<LOQ	<LOQ	<LOQ

Supplementary table S6. Average relative abundances (%) of the bacterial families in the mucosal environment in the different test conditions (Ctrl= control, CG= chitin-glucan, CGB= chitin-glucan + B. breve) after 48h. Statistically significant differences are indicated in bold. The intensity of shading is correlated with the abundance of a given family under different conditions per donor. Significant differences reported in the tables (bold values) refer to differences CG versus the control condition and to CG versus CGB.

Phylum	Family	Mucus								
		Donor 1			Donor 2			Donor 3		
		Ctrl	CG	CGB	Ctrl	CG	CGB	Ctrl	CG	CGB
Actinobacteria	*Atopobiaceae*	<LOQ	<LOQ	<LOQ	<LOQ	<LOQ	<LOQ	0,02	0,04	0,03
	Bifidobacteriaceae	0,80	1,05	1,10	0,01	0,01	0,02	6,29	6,88	5,75
	Coriobacteriaceae	0,24	0,61	0,49	3,73	4,55	3,04	0,59	0,76	1,04
	Coriobacteriales Incertae Sedis	<LOQ	<LOQ	<LOQ	<LOQ	<LOQ	<LOQ	0,00	<LOQ	0,00
	Coriobacteriales unclassified	<LOQ	<LOQ	<LOQ	<LOQ	<LOQ	<LOQ	0,00	0,00	0,00
	Eggerthellaceae	0,02	0,06	0,02	0,01	0,02	**0,00**	0,04	0,08	0,08
Bacteroidetes	*Bacteroidaceae*	11,57	14,75	11,27	25,28	28,03	25,60	5,52	3,11	2,77
	Bacteroidales unclassified	0,00	0,00	0,00	<LOQ	<LOQ	<LOQ	0,00	0,00	<LOQ
	Bacteroidia unclassified	0,01	0,05	0,01	<LOQ	<LOQ	<LOQ	0,01	0,02	0,02
	Barnesiellaceae	0,00	0,02	0,01	0,01	0,01	0,00	<LOQ	<LOQ	**0,01**
	Marinifilaceae	0,03	0,08	0,02	0,06	**0,03**	0,03	0,01	0,01	**0,02**
	Muribaculaceae	<LOQ	<LOQ	<LOQ	<LOQ	<LOQ	<LOQ	0,01	0,04	0,03
	Prevotellaceae	<LOQ	<LOQ	0,00	<LOQ	<LOQ	<LOQ	0,01	0,15	0,17
	Rikenellaceae	0,03	0,26	0,07	0,44	0,23	0,18	0,17	0,16	0,28
	Tannerellaceae	0,30	0,91	0,51	0,38	0,64	0,39	0,74	1,21	1,47
Firmicutes	*Acidaminococcaceae*	0,57	0,88	0,61	1,32	0,52	0,79	0,00	<LOQ	0,00
	Anaerovoracaceae	0,00	0,00	<LOQ	<LOQ	<LOQ	0,00	0,02	0,04	0,02
	Butyricicoccaceae	0,01	0,05	0,01	2,76	1,45	3,16	0,05	0,02	0,03
	Christensenellaceae	0,00	0,00	0,00	0,00	<LOQ	<LOQ	0,01	0,03	0,03
	Clostridia UCG-014	0,00	0,00	0,00	<LOQ	<LOQ	<LOQ	0,03	0,07	0,07
	Clostridia unclassified	<LOQ	0,00	0,00	0,01	0,04	0,03	<LOQ	<LOQ	<LOQ
	Clostridia vadinBB60 group	<LOQ	<LOQ	<LOQ	<LOQ	<LOQ	<LOQ	0,00	<LOQ	0,00
	Clostridiaceae	0,37	3,13	2,78	14,39	23,61	17,74	3,39	2,30	2,02
	Enterococcaceae	0,73	1,14	1,10	0,03	0,04	0,03	<LOQ	0,00	0,00
	Erysipelatoclostridiaceae	0,01	0,10	0,02	0,10	**0,37**	0,23	0,00	0,02	0,01
	Erysipelotrichaceae	0,01	0,10	0,03	0,02	0,04	0,02	2,80	4,63	3,92
	Eubacteriaceae	0,00	**0,03**	0,02	<LOQ	<LOQ	<LOQ	0,05	0,03	0,04
	Lachnospiraceae	60,75	52,76	47,84	50,50	39,20	47,16	74,69	76,24	77,09
	Lactobacillaceae	<LOQ	<LOQ	<LOQ	<LOQ	<LOQ	0,00	<LOQ	<LOQ	<LOQ

	Lactobacillales unclassified	0,00	0,01	0,01	<LOQ	<LOQ	<LOQ	<LOQ	<LOQ	<LOQ
	Monoglobaceae	0,00	0,01	0,00	<LOQ	<LOQ	<LOQ	0,02	0,02	0,00
	Oscillospiraceae	0,26	0,53	0,34	0,34	0,30	0,70	0,74	0,85	0,91
	Oscillospirales	<LOQ	<LOQ	<LOQ	<LOQ	0,00	<LOQ	0,00	0,00	0,00
	Oscillospirales unclassified	<LOQ	<LOQ	<LOQ	<LOQ	<LOQ	<LOQ	0,00	<LOQ	<LOQ
	Peptococcaceae	<LOQ	0,00	0,00	<LOQ	<LOQ	<LOQ	<LOQ	<LOQ	<LOQ
	Peptostreptococcaceae	0,01	0,07	0,03	0,00	0,00	0,01	0,03	0,02	**0,07**
	RF39	<LOQ	<LOQ	<LOQ	<LOQ	<LOQ	<LOQ	0,00	<LOQ	0,00
	Ruminococcaceae	0,30	0,69	0,56	0,07	0,07	0,04	0,69	1,07	1,02
	Streptococcaceae	0,00	0,02	0,00	0,00	<LOQ	<LOQ	<LOQ	0,00	0,00
	UCG-010	<LOQ	<LOQ	<LOQ	<LOQ	<LOQ	<LOQ	<LOQ	<LOQ	0,00
	Veillonellaceae	0,01	0,04	0,03	0,11	0,06	0,15	0,29	0,27	0,30
Verrucomicrobia	*vadinBE97*	<LOQ	<LOQ	0,00	<LOQ	<LOQ	<LOQ	0,00	<LOQ	0,00
Proteobacteria	*Alphaproteobacteria unclassified*	<LOQ	<LOQ	<LOQ	<LOQ	<LOQ	<LOQ	0,00	0,00	0,01
	Desulfovibrionaceae	0,02	0,02	0,00	0,02	0,02	0,02	0,00	0,00	0,00
	Enterobacterales unclassified	0,00	0,00	0,01	<LOQ	<LOQ	<LOQ	<LOQ	<LOQ	<LOQ
	Enterobacteriaceae	23,90	22,59	33,03	0,39	0,76	0,65	3,76	1,85	2,71
	Gammaproteobacteria unclassified	0,00	0,00	0,00	<LOQ	<LOQ	<LOQ	<LOQ	<LOQ	<LOQ
	Sutterellaceae	0,00	0,03	0,01	0,02	0,01	0,01	0,02	0,04	0,05
Unclassified	*Bacteria unclassified*	0,03	0,00	0,03	<LOQ	<LOQ	<LOQ	<LOQ	<LOQ	<LOQ

Supplementary table S7. Average relative abundances (%) of the most abundant OTUs in the mucosal environment in the different test conditions (Ctrl= control, CG= chitin-glucan, CGB= chitin-glucan + B. breve) after 48h. Statistically significant differences are indicated in bold. The intensity of shading is correlated with the abundance of a given family under different conditions per donor. Significant differences reported in the tables (bold values) refer to differences CG versus the control condition and to CG versus CGB.

Phylum	Family	OTU	Closely related species	Mucus								
				Donor 1			Donor 2			Donor 3		
				Ctrl	CG	CGB	Ctrl	CG	CGB	Ctrl	CG	CGB
Actinobacteria	*Bifidobacteriaceae*	21	*Bifidobacterium bifidum*	<LOQ	<LOQ	0,00	<LOQ	<LOQ	<LOQ	4,83	5,62	4,62
		34	*Bifidobacterium adolescentis, B. faecale*	0,00	**0,01**	**0,00**	0,00	0,00	0,01	0,25	0,25	0,23
		37	*Bifidobacterium longum subsp. null/longum*	0,70	0,96	0,97	0,00	0,00	0,00	1,11	0,92	0,84
		211	*Bifidobacterium breve*	<LOQ	<LOQ	0,00	<LOQ	0,00	0,00	<LOQ	<LOQ	<LOQ
	Coriobacteriaceae	14	*Collinsella aerofaciens, Coriobacterium sp.*	0,13	0,32	0,22	3,73	4,55	3,04	0,59	0,76	1,04
Bacteroidetes	*Bacteroidaceae*	4	*Bacteroides sp.*	1,16	1,10	0,94	17,70	17,77	19,15	0,57	0,24	0,16
		8	*Bacteroides uniformis*	0,12	**0,41**	0,27	0,90	**2,74**	**1,47**	0,01	0,01	0,01
		11	*Bacteroides uniformis*	0,25	**1,08**	0,64	0,16	**0,49**	**0,25**	0,36	0,29	0,27
		13	*Bacteroides vulgatus*	0,89	1,40	0,92	0,59	**0,19**	0,21	0,72	0,52	0,33
		16	*Bacteroides massiliensis*	1,83	3,06	**1,50**	0,12	**0,04**	0,04	1,60	0,93	1,18
		17	*Bacteroides caccae*	2,17	1,66	1,61	2,59	2,89	1,83	1,12	**0,42**	0,30
		22	*Bacteroides ovatus*	0,13	0,16	0,11	1,78	2,79	1,90	0,35	0,23	0,18
		24	*Bacteroides stercoris(98)*	2,99	3,68	3,33	<LOQ	<LOQ	0,00	0,31	0,18	0,17
		25	*Bacteroides eggerthii*	1,51	1,82	1,46	0,96	0,46	0,24	<LOQ	<LOQ	<LOQ
	Rikenellaceae	47	*Alistipes finegoldii, A. onderdonkii, Bacteroides sp.*	0,01	0,07	0,02	0,37	0,13	0,09	0,15	0,11	0,23
	Tannerellaceae	12	*Parabacteroides distasonis*	0,02	0,09	0,03	0,06	0,16	0,10	0,56	1,04	1,23
		35	*Parabacteroides merdae*	0,23	0,48	0,34	0,30	0,47	0,27	0,15	0,14	0,21
Firmicutes	*Acidaminococcaceae*	29	*Phascolarctobacterium faecium*	0,57	0,88	0,61	1,32	0,52	0,79	0,00	<LOQ	0,00
	Clostridiaceae	6	*Clostridium butyricum*	0,08	1,92	2,33	14,35	23,52	17,67	<LOQ	0,00	<LOQ
		31	*Clostridium tertium , C. chauvoei*	0,28	0,43	0,37	<LOQ	<LOQ	0,01	3,33	2,24	1,90
	Clostridiaceae	32	*Butyricicoccus sp.(96)*	<LOQ	0,00	0,00	2,76	1,45	3,15	0,02	0,00	0,00
		106	*Clostridium neonatale*	<LOQ	0,71	0,03	<LOQ	<LOQ	<LOQ	<LOQ	<LOQ	<LOQ
	Enterococcaceae	10	*Enterococcus faecium, E. durans, E. hirae, E. azikeevi, E. villorum*	0,72	1,13	1,08	0,03	0,04	0,03	<LOQ	0,00	0,00
	Erysipelotrichaceae	26	*Holdemanella sp.*	<LOQ	<LOQ	<LOQ	0,01	0,04	0,02	2,69	4,48	3,76
	Lachnospiraceae	1	*Roseburia hominis, R. intestinalis*	38,78	26,93	23,42	7,71	3,82	3,67	21,84	23,66	21,06
		2	*Roseburia inulinivorans*	10,78	11,68	9,19	31,22	22,33	29,09	36,40	32,17	38,27
		5	*Eubacterium rectale, Clostridiales bacterium*	0,05	0,08	0,09	8,11	8,84	8,65	11,57	14,56	12,22
		7	*butyrate-producing bacterium L2-12, Eubacterium ventriosum*	0,04	**0,58**	0,49	0,36	**0,93**	**1,39**	0,02	**0,46**	**0,33**
		9	*Lachnospiraceae_NK4A136_group(93)*	6,98	**10,15**	9,80	0,37	0,40	0,71	0,68	**1,14**	1,41
		15	*Dorea longicatena*	0,10	0,20	0,25	0,18	0,24	0,33	0,83	0,98	0,78
		28	*Ruminococcus torques*	1,65	0,65	1,13	0,79	0,67	0,65	0,74	**0,43**	0,48
		30	*Blautia obeum*	0,11	0,14	0,18	0,00	0,01	0,01	0,38	0,45	0,48
		42	*Ruminococcus faecis, Clostridiales bacterium(99), R. torques*	0,20	0,26	0,50	0,01	0,01	0,02	0,10	**0,01**	0,03
		48	*Clostridium aldenense*	0,01	0,01	0,01	0,18	0,19	0,56	0,02	0,02	0,02
		49	*Butyrate-producer, Coprococcus comes*	0,20	0,22	0,38	0,04	0,05	0,06	0,06	0,09	0,05
		50	*Member of Lachnospiraceae*	0,11	0,22	0,29	0,20	0,25	0,33	0,04	0,05	0,03
		70	*Lachnospiraceae incertae sedis (96)*	0,00	0,00	0,01	0,28	0,28	0,30	0,00	<LOQ	0,00
		78	*Member of Lachnospiraceae*	0,00	0,02	0,03	0,04	0,02	**0,05**	0,31	0,37	0,36
		87	*Member of Clostridium cluster XIVa*	0,32	0,25	0,22	<LOQ	0,00	<LOQ	0,09	0,01	0,01
	Ruminococcaceae	20	*Faecalibacterium prausnitzii*	0,27	0,60	0,48	0,05	0,06	0,03	0,38	0,62	0,47
		76	*Member of Ruminococcaceae*	0,00	0,00	0,00	0,15	0,15	0,33	0,21	0,22	0,24
	Veillonellaceae	38	*Dialister sp.*	<LOQ	<LOQ	<LOQ	0,01	0,00	0,01	0,28	0,27	0,29
Proteobacteria	*Enterobacteriaceae*	3	*Escherichia coli, Shigella sonnei, E. vulneris, Photorhabdus luminescens*	23,86	22,57	32,99	0,39	0,76	0,65	3,72	1,81	2,67

Supplementary figures

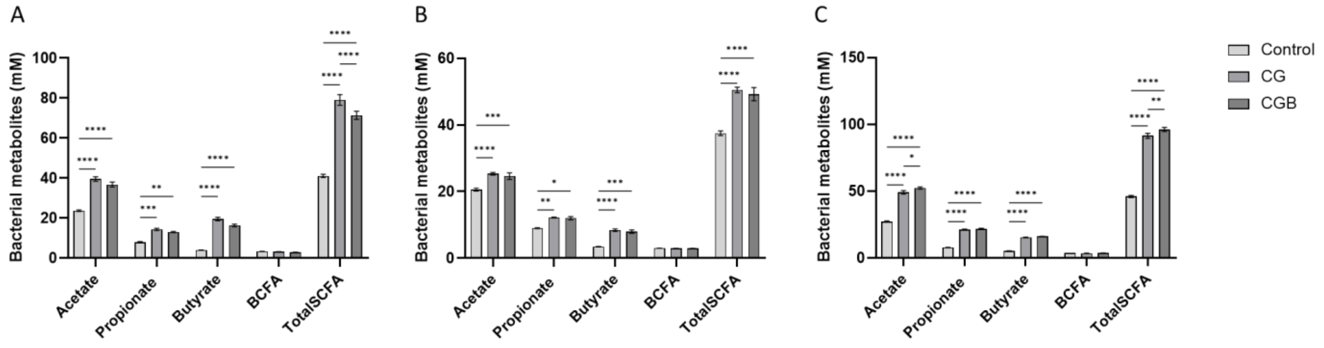

Supplementary figure S1. Effect of CG and CGB on SCFA and BCFA in different donors. (A), (B) and (C) represent different bacterial metabolites (Δ48h) in control, CG and CGB treated reactors (mean ±SEM, n=3) for donor 1, 2 and 3, respectively. Significant differences between treatments are marked with asterisks (p<0.05, ** p<0.01, *** p<0.001, **** p <0.0001).*

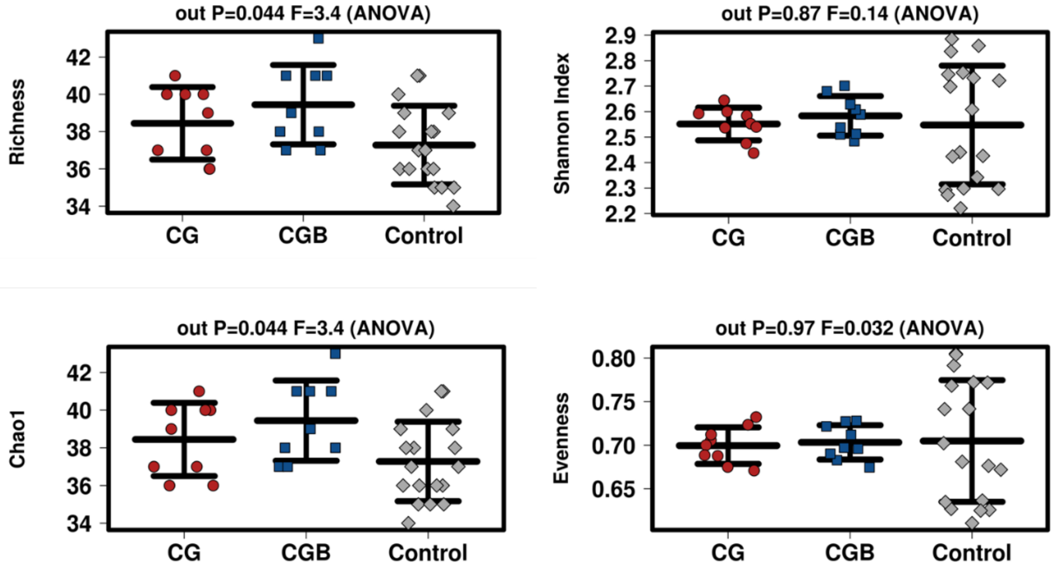

Supplementary figure S2. Alpha diversity, richness, and evenness indices in the luminal compartment of CG, CGB and control reactors. Strip charts shows richness, Shannon Index, Chao1 and Evenness indices calculated from 16S rRNA sequencing data at OTU level from three healthy individuals (n=3) and three technical replicas (N=3)

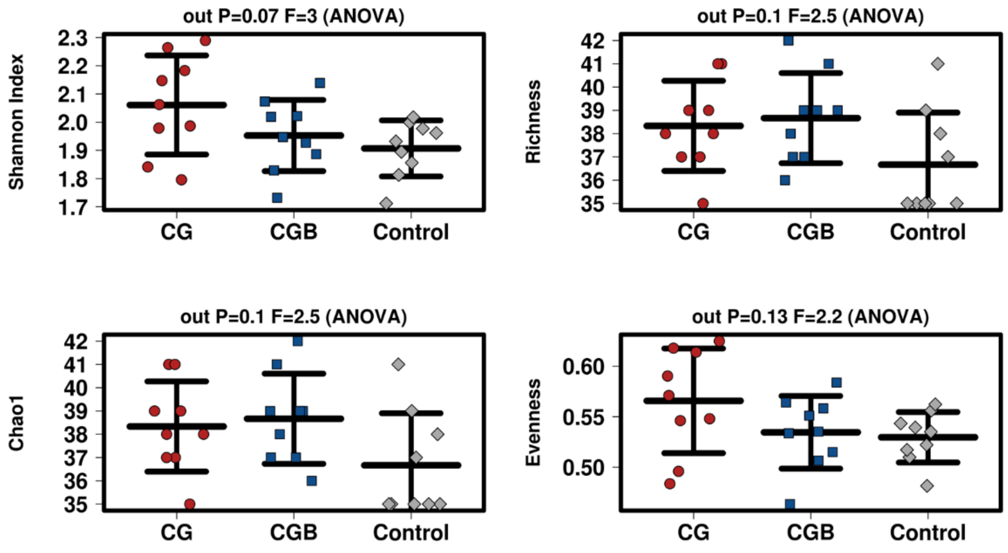

Supplementary figure S3. Alpha diversity, richness, and evenness indices in the mucosal compartment of CG, CGB and control reactors. Strip charts shows richness, Shannon Index, Chao1 and Evenness indices calculated from 16S rRNA sequencing data at OTU level from three healthy individuals (n=3) and three technical replicas (N=3)

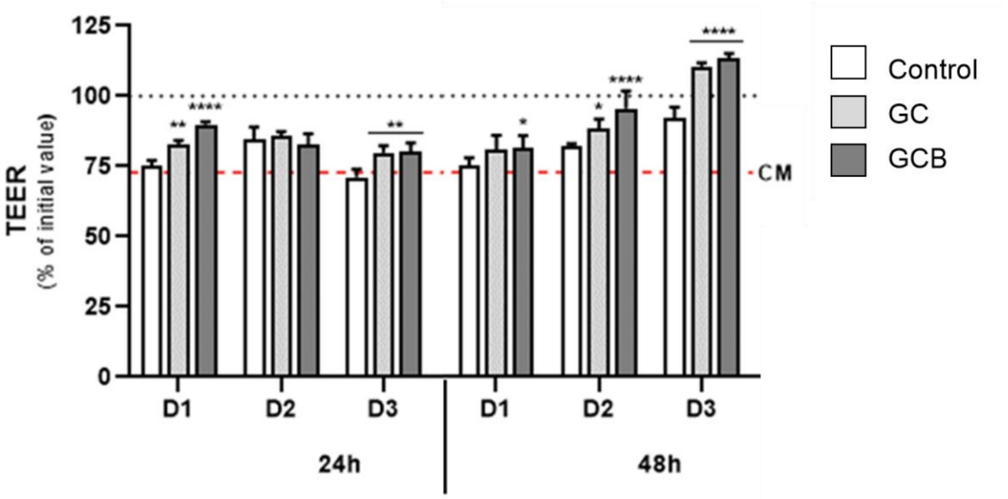

Supplementary figure S4. Interindividual differences of TEER in response to CG and CGB. Bars represent the mean ± SEM of n = 3. CM = cell culture media;. Significant differences are represented by (*). (*), (**), (***) and (****) represent p<0.05, p<0.01, p<0.001 and p<0.0001, respectively.

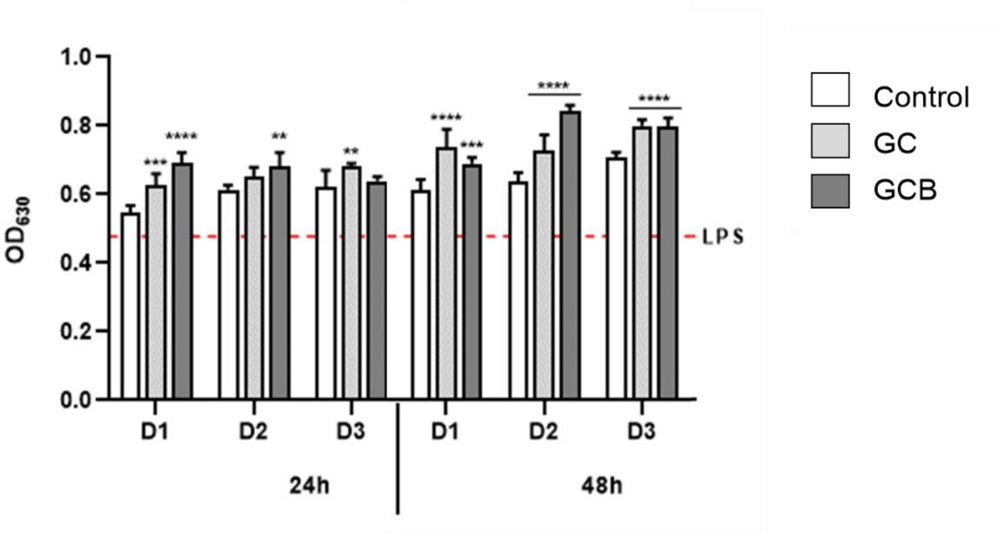

Supplementary figure S5. Interindividual differences of NFkβ in response to CG and CGB. Bars represent the mean ± SEM of n = 3. LPS = lipopolysaccharide. Significant differences are represented by (*). (*), (**), (***) and (****) represent p<0.05, p<0.01, p<0.001 and p<0.0001, respectively.

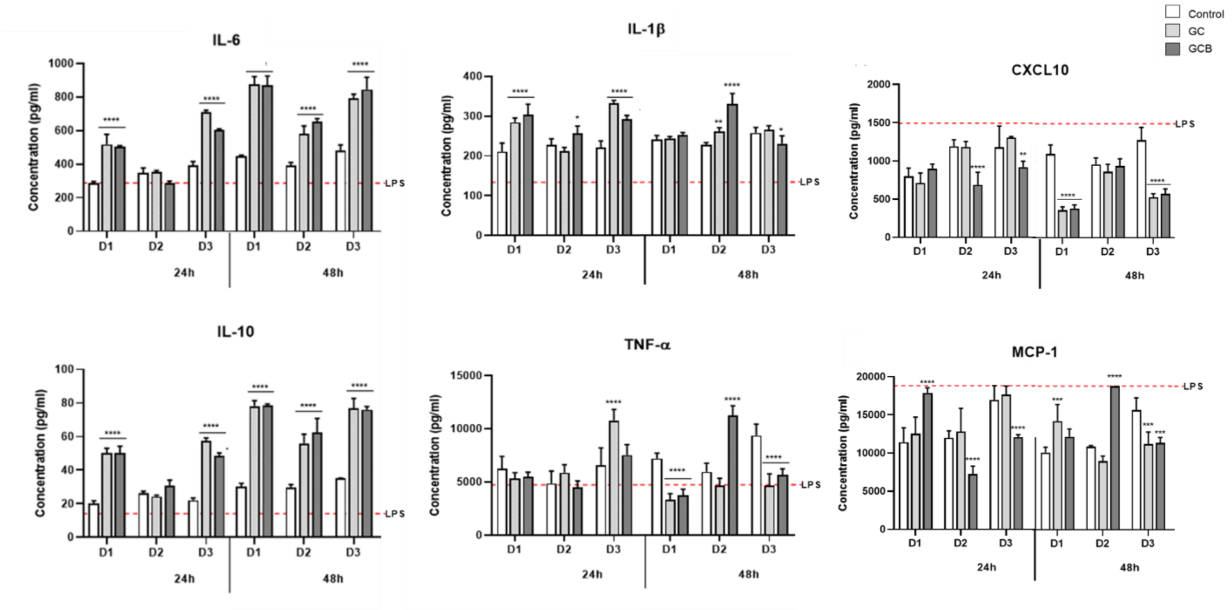

Supplementary figure S6. Interindividual differences of cytokine production in response to CG and CGB. Bars represent the mean ± SEM of n = 3. LPS = lipopolysaccharide. Significant differences are represented by (*). (*), (**), (***) and (****) represent p<0.05, p<0.01, p<0.001 and p<0.0001, respectively.

References supplementary material

Lopez-Siles, M., Martinez-Medina, M., Busquets, D., Sabat-Mir, M., Duncan, S. H., Flint, H. J., Aldeguer, X., & Garcia-Gil, L. J. (2014). Mucosa-associated Faecalibacterium prausnitzii and Escherichia coli co-abundance can distinguish Irritable Bowel Syndrome and Inflammatory Bowel Disease phenotypes. *International Journal of Medical Microbiology*, *304*(3), 464-475. https://doi.org/https://doi.org/10.1016/j.ijmm.2014.02.009

Rinttilä, T., Kassinen, A., Malinen, E., Krogius, L., & Palva, A. (2004). Development of an extensive set of 16S rDNA-targeted primers for quantification of pathogenic and indigenous bacteria in faecal samples by real-time PCR. *Journal of Applied Microbiology*, *97*(6), 1166-1177. https://doi.org/https://doi.org/10.1111/j.1365-2672.2004.02409.x
